# Predominance of Rotavirus P[4]G2 in a Vaccinated Population, Brazil

**DOI:** 10.3201/eid1310.070412

**Published:** 2007-10

**Authors:** Ricardo Q. Gurgel, Luis E. Cuevas, Sarah C.F. Vieira, Vanessa C.F. Barros, Paula B. Fontes, Eduardo F. Salustino, Osamu Nakagomi, Toyoko Nakagomi, Winifred Dove, Nigel Cunliffe, Charles A. Hart

**Affiliations:** * Federal University of Sergipe, Aracaju, Brazil; † University of Liverpool, Liverpool, United Kingdom; ‡ Liverpool School of Tropical Medicine, Liverpool, United Kingdom; § Nagasaki University, Nagasaki, Japan

**Keywords:** Rotavirus, genotypes, diarrhea, Brazil, vaccine, dispatch

## Abstract

We identified 21 rotaviruses in 129 patients with diarrhea in a Brazilian city with high rotavirus vaccine coverage. All rotaviruses were genotype P[4]G2 with 1 mixed infection with P[NT]G9. Although virus predominance could have occurred randomly, the vaccine may be less protective against P[4]G2. Prospective surveillance is urgently needed.

Rotavirus causes severe diarrhea, illness, and death worldwide (*1*). Infection rates with rotavirus remain high despite improved sanitation, and vaccination is likely to be the best control strategy (*2*,*3*). Several candidate vaccines are being developed, 2 are already licensed (*4*,*5*) and at least 5 are being evaluated (*2*,*6*). The 2 licensed rotavirus vaccines are designed to provide protection against rotavirus gastroenteritis caused by the most common worldwide circulating rotavirus serotypes (*2*). These include G types G1, G2, G3, and G4 and P types P[4] and P[8] (on the basis of variability in the outer capsid proteins VP7 and VP4, respectively).

One of the currently licensed vaccines (Rotarix; GlaxoSmithKline, Rixensart, Belgium), a live, attenuated, human monovalent rotavirus P[8]G1 vaccine, was highly efficacious for preventing severe rotavirus gastroenteritis in phase III efficacy studies in Latin America and Europe. These studies included Brazil, where the main circulating rotavirus genotypes were P[8]G1 and P[8]G9 (*5*). Brazil therefore took the unprecedented step of introducing this vaccine into its national Expanded Program for Immunization in March 2006 and provided 2 free doses to all children <3 months of age. Vaccination coverage among eligible age cohorts has increased and reached 51% in Sergipe (www.datasus.gov.br) in northeastern Brazil. However, this vaccine appears less effective in preventing severe rotavirus gastroenteritis caused by P[4]G2 strains (*5*), and immunologic pressure exerted by the vaccine may cause emergence of rotavirus genotypes that are not controlled by the vaccine. This possibility could change the pattern and distribution of the most prevalent rotavirus strains in the vaccinated population.

Because this is an unprecedented epidemiologic situation, we monitored the effect the vaccine might have on predominant genotypes. We describe rotavirus genotypes recovered from children with acute diarrhea in Aracaju, Sergipe, Brazil, after the widespread introduction of the vaccine.

## The Study

Children with acute diarrhea who came to 2 public hospitals (Joao Alves Filho and Municipal da Zona Norte) and 3 health centers that provided health services to a population (Santa Maria) in Aracaju, Brazil, were enrolled from November 2006 to February 2007. Children who came to the hospitals were enrolled consecutively on specific days of the week by study health workers, and children who came to the health centers were visited at home after we checked the daily attendance lists of the centers. Acute diarrhea was defined as any episode <14 days duration with >3 watery stools per day. Background and clinical information were collected after obtaining parental consent, and stool samples were stored frozen in duplicate at –80°C until analyzed in Liverpool, UK. Information on rotavirus vaccination was obtained from parents and cross-checked against vaccination record cards. A child was considered vaccinated if 2 doses of the vaccine had been recorded on the vaccination card. Rotavirus detection, genotyping, electropherotyping, isolation of strains in cell culture, and sequencing were performed as described (*7*). Severity of diarrhea episodes was classified according to a modified Vesikari score (*8*). Data were analyzed by using descriptive statistics in Epi-Info 2002 (Centers for Disease Control and Prevention, Atlanta, GA, USA). The study protocol was reviewed and approved by the Ethics Committees of the Liverpool School of Tropical Medicine and the Federal University of Sergipe.

A total of 129 patients with a median age of 12 months (range 1 month–12 years) were enrolled. Of these, 63 (49%) were <1 year of age, 39 (30%) were 1–2 years of age, and 27 (21%) were >2 years of age. A total of 21 children (16%) were positive for rotavirus by ELISA. Of these children, 20 were identified among 89 children enrolled in the hospital and 1 was identified among 40 children enrolled in the health centers (p = 0.002). Forty-eight children (37%) had received the rotavirus vaccine. The frequency of rotavirus infection by vaccination status and age is shown in the Table. Among children <1 year of age, 3 (7%) of the 44 vaccinated children were infected with rotavirus compared with 5 (26%) of 19 children who did not receive the vaccine (p<0.05). Among children 1–2 years of age, 4 had received the vaccine and 1 (25%) of them was infected with rotavirus compared with 8 (23%) of the 35 children who did not receive the vaccine (p not significant).

**Table:** Frequency of rotavirus infection by vaccination status and age, Aracaju, Sergipe, Brazil*

Age, y	Rotavirus	Vaccinated, no. (%)	Not vaccinated, no. (%)	p value
<1	Positive	3 (7)	5 (26)	0.05
	Negative	41 (93)	14 (74)	
1–2	Positive	1 (25)	8 (23)	NS
	Negative	3 (75)	27(77)	
>2	Positive	NA	4 (15)	
	Negative	NA	23 (85)	

*Children were considered vaccinated if they had received 2 doses of vaccine. NS, not significant; NA, not applicable.

The median (range) diarrhea severity scores of children with and without rotavirus infection were 12.9 (10–15.8) and 9.4 (5.3–13.5), respectively (p<0.001). Although numbers are small, vaccinated children had a median (range) diarrhea severity scores of 12.5 (7–15) if they were infected with rotavirus and 7 (3–17) if they were not infected. Similarly, the median (range) severity scores for unvaccinated children were 13 (8–15) and 11 (4–14) for children with and without rotavirus infections, respectively (p not significant).

All 21 rotavirus infections were with genotype P[4]G2. One child had a mixed infection with P[4]G2 and P[NT]G9. Nineteen specimens had short electropherotype strains, 1 was positive but with an undefined pattern, and 1 had insufficient RNA to produce a pattern.

## Conclusions

Sergipe has achieved relatively high rotavirus vaccine coverage (54%) since introduction of the vaccine in 2006, with 48,165 doses provided in Aracaju. The vaccine was well received by the local population, and as new eligible children continue to be vaccinated, it is likely that vaccination levels will reach the high coverage currently attained for oral polio vaccine (100%) (http://tabnet.datasus.gov.br/cgi/tabcgi.exe?idb2005/f13.def).

To our knowledge, this is the first report from Brazil of 1 rotavirus genotype predominating in a population after introduction of a vaccine. The P[4]G2 strain is a genotype for which effectiveness of the vaccine appears to be lower. This genotype has been previously reported in Brazil but represents only 6.1% of all the genotypes published since 2000. The proportions of strains with P[4]G2 has ranged from 0% to 27% in various studies, and no study reported that this was the predominant strain. Our finding of 100% prevalence of this genotype is unusual. Limited evidence of the effectiveness of Rotarix vaccine against the P[4]G2 strain has been reported (*9**,**10*) because the VP4 and VP7 proteins are not found in the P[8]G1 strain that is included in this vaccine.

Although our numbers are small, a lower proportion of vaccinated children had rotavirus-associated diarrhea, which likely reflects the protective effect of the vaccine. Four children were infected despite having been vaccinated and their infections were as severe as those in children who had not received the vaccine. This finding confirms that the vaccine does not afford complete protection against infection. Although predominance of the P[4]G2 strain in this population could be due to random preponderance of this genotype and is unrelated to vaccine use, this epidemiologic finding highlights the need for postlicensure surveillance of the vaccinated population.

## References

[1] Parashar UD, Hummelman EG, Bresee JS, Miller MA, Glass RI. Global illness and deaths caused by rotavirus disease in children. Emerg Infect Dis. 2003;9:565–72. 12737740 10.3201/eid0905.020562 PMC2972763

[2] Glass RI, Parashar UD, Bresee JS, Turcios R, Fischer TK, Widdowson MA, Rotavirus vaccines: current prospects and future challenges. Lancet. 2006;368:323–32. 10.1016/S0140-6736(06)68815-6 16860702

[3] Oliveira CS, Linhares AC. Rotavirus: clinical features and prevention. J Pediatr (Rio J). 1999;75(Suppl 1):S91–102. 14685487 10.2223/jped.376

[4] Vesikari T, Matson DO, Dennehy P, Van Damme P, Santosham M, Rodriguez Z, Safety and efficacy of a pentavalent human-bovine (WC3) reassortant rotavirus vaccine. N Engl J Med. 2006;354:23–33. 10.1056/NEJMoa052664 16394299

[5] Ruiz-Palacios GM, Perez-Schael I, Velazquez FR, Abate H, Breuer T, Clemens SC, Safety and efficacy of an attenuated vaccine against severe rotavirus gastroenteritis. N Engl J Med. 2006;354:11–22. 10.1056/NEJMoa052434 16394298

[6] Dennehy PH. Rotavirus vaccines—an update. Vaccine. 2007;25:3137–41. 10.1016/j.vaccine.2007.01.102 17321017

[7] Montenegro FM, Correia JB, Rodrigues Falbo A, Dove W, Nakagomi T, Nakagomi O, Anticipating rotavirus vaccines in Brazil: detection and molecular characterization of emerging rotavirus serotypes G8 and G9 among children with diarrhoea in Recife, Brazil. J Med Virol. 2007;79:335–40. 10.1002/jmv.20803 17245712

[8] Nakagomi T, Nakagomi O, Takanashi Y, Enoki M, Suzuki T, Kilgore PE. Incidence and burden of rotavirus gastroenteritis in Japan, as estimated from a prospective sentinel hospital study. J Infect Dis. 2005;192(Suppl 1):S106–10. 10.1086/431503 16088792

[9] Bernstein DI. Live attenuated human rotavirus vaccine, Rotarix. Semin Pediatr Infect Dis. 2006;17:188–94. 10.1053/j.spid.2006.08.006 17055369

[10] Rahman M, Sultana R, Ahmed G, Nahar S, Hassan ZM, Saiada F, Prevalence of G2P[4] and G12P[6] rotavirus, Bangladesh. Emerg Infect Dis. 2007;13:18–24. 10.3201/eid1301.060910 17370511 PMC2725799

